# Seroprevalence and risk factors for *Coxiella burnetii*, the causative agent of Q fever in the dromedary camel (*Camelus dromedarius*) population in Algeria

**DOI:** 10.4102/ojvr.v84i1.1461

**Published:** 2017-08-31

**Authors:** Mohammed H. Benaissa, Samir Ansel, Abdallah Mohamed-Cherif, Karima Benfodil, Djamel Khelef, Curtis R. Youngs, Rachid Kaidi, Khatima Ait-Oudhia

**Affiliations:** 1 Scientific and Technical Research Centre for Arid Areas (CRSTRA), Touggourt, Algeria; 2 Higher National Veterinary School, Algiers, Algeria; 3 Animal Science Department, Iowa State University, United States; 4 Institute of Veterinary Sciences, LBRA, University of Blida, Algeria

## Abstract

Query (Q) fever is a globally distributed zoonotic disease caused by *Coxiella burnetii*, a bacterial agent for which ruminants are the most prevalent natural reservoir. Data regarding Q fever infection in camels in Algeria are limited. Therefore, a survey to detect seroprevalence of *C. burnetii* antibodies was conducted among healthy camel populations in a vast area in southeastern Algeria to determine distribution of the Q fever causative organism and to identify risk factors associated with infection. Between January and March 2016, blood samples were collected from 184 camels and serum samples were subsequently analysed using a commercial Enzyme-Linked Immunosorbent Assay (ELISA) kit. At the time of blood collection, a questionnaire investigating 13 potential predisposing factors associated with *C. burnetii* seropositivity was completed for every dromedary camel and herd. Results were analysed by a chi-square (χ^2^) test and multivariate logistic regression. The seroprevalence of *C. burnetii* at the animal level was 71.2% (95% CI: 65.2–78.3) and 85.3% (95% CI: 72.8–97.8) at the herd level. At the animal level, differences in seroprevalence were observed because of herd size, animal age, animal sex, presence of ticks and contact with other herds. A multivariable logistic regression model identified three main risk factors associated with individual seropositivity: (1) age class > 11 years (OR = 8.81, 95% CI: 2.55–30.41), (2) herd size > 50 head (OR = 4.46, 95% CI: 1.01–19.59) and (3) infestation with ticks (OR 2.2; 95% CI: 1.1–4.5). This study of seroprevalence of *C. burnetii* infection in camels in Algeria revealed a high seroprevalence of Q fever in camel populations in southeastern Algeria and provided strong evidence that Q fever represents an economic, public health and veterinary concern. Appropriate measures should be taken to prevent the spread of *C. burnetii* and to reduce the risk of Q fever in farm animals and humans in this agro-ecologically and strategically important region of North Africa.

## Introduction

Query (Q) fever is a zoonotic disease with worldwide distribution with the exception of New Zealand. *Coxiella burnetii*, the causative agent of Q fever, is an obligate intracellular bacterium. Recently, this bacterium was classified into the Legionellales order and the Coxiellaceae family (Bielawska-Drózd et al. 2013). Infection with *C. burnetii* has been detected in humans and a wide range of animal species (Cutler, Bouzid & Cutler 2007).

The economic and public health impacts of Q fever remain a major concern in developing countries because Q fever causes significant loss of animal productivity and is a zoonotic risk to humans (Mostafavi et al. 2012; Van Asseldonk et al. 2015). In many livestock species, Q fever is frequently asymptomatic. Clinical expression of *C. burnetii* infection in sheep and goats, however, includes late gestation abortion, reduced reproductive efficiency because of stillbirths, delivery of weak offspring and premature delivery (Angelakis & Raoult 2010), whereas cattle may develop metritis, mastitis and infertility (To et al. 1998). Domestic ruminants are considered the principle reservoirs for this infectious agent and are frequently incriminated as sources of Q fever outbreaks in humans (Alvarez et al. 2012; Eldin et al. 2017; Vanderburg et al. 2014). Ticks are also considered a natural reservoir of *C. burnetii* (De Bruin et al. 2013).

*Coxiella burnetii* is transmitted to humans through direct contact with milk, urine, faeces, amniotic fluid or aborted tissues and placentae at birth (EFSA 2012). Because *C. burnetii* is a highly resistant bacterium, the environment itself can serve as a reservoir (De Bruin et al. 2013). Inhalation of aerosolised particles from live ruminants and aborted foetuses is a major source of infection for humans (Isken et al. 2013).

Numerous seroprevalence surveys of *C. burnetii* infection in camels have been conducted across the globe, including the countries of Tunisia (Burgemeister, Leyk & Goessler 1975), Chad (Schelling et al. 2003), Saudi Arabia (Hussein et al. 2008, 2015; Mohammed et al. 2014), Kenya (Browne et al. 2017), United Arab Emirates (Afzal & Sakkir 1994) and Iran (Doosti, Arshi & Sadeghi 2014; Mostafavi et al. 2012; Pirouz et al. 2015). These surveys revealed that Q fever seroprevalence varies widely by animal species and geographical location. Risk factors underlying this variability in infection rate are poorly understood (Vanderburg et al. 2014). Previous studies have established that camels can harbour high concentrations of *C. burnetii* (Mohammed et al. 2014).

In Algeria, Q fever is considered an endemic infection. Interestingly, very few studies have documented the seroprevalence of Q fever in Algerian farm animals and most investigations have focused on sheep and goats (Khaled et al. 2016; Rahal et al. 2011; Yahiaoui et al. 2013). The first published case of human coxiellosis in Algeria dates back to 1948 (Pierrou et al. 1956), but since then few epidemiological surveys have been published (Lacheheb & Raoult 2009). A limited number of human cases of coxiellosis have been reported in Algeria and most cases occurred in the northern part of the country (Angelakis et al. 2014; Benslimani et al. 2005). More recently, evidence of Q fever infection based on polymerase chain-reaction (PCR) amplification and sequencing of *C. burnetii* target genes has been reported in ticks from bats (Leulmi et al. 2016), *Rhipicephalus bursa* ticks, dog and cat spleens (Bessas et al. 2016) and blood from sheep and goats (Aouadi et al. 2017). The true incidence of the disease in humans remains unknown in Algeria because of a lack of published data and the non-specific clinical signs of Q fever which lead to underreporting of the disease (Van der Hoek et al. 2012).

In southern Algeria, camels play multiple roles in the agricultural economy. Most camels are reared using traditional husbandry practices that typically are characterised by very poor hygienic conditions. The re-emergence of Q fever infections worldwide, coupled with the scarcity of information on the status of camel coxiellosis in Algeria, led us to investigate the epidemiological situation of *C. burnetii* seropositivity at the individual and herd level as well as to determine the distribution of and risk factors associated with this infection in camel herds in southeast Algeria.

## Materials and methods

### Study area

**FIGURE 1:**
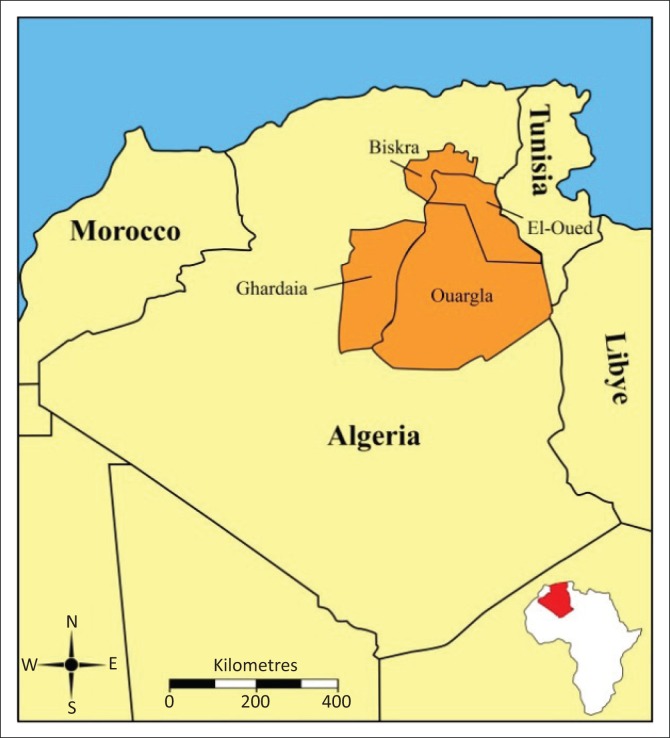
Map of Algeria (highlighted in red in inset) depicting the four study provinces (burnt orange colour).

This study was carried out in four provinces (Biskra, El-Oued, Ouargla and Ghardaia) in southeastern Algeria. These provinces are located at 002° 04 to 007° 35 E and 28° 32 to 34° 56 N (Figure 1). This region is considered one of the most significant camel rearing areas in Algeria where camel milk is becoming increasingly commercialised and consumed. The climate of this province is arid and is characterised by long, hot summers and short winters.

### Sampling procedure

The sample size necessary for detection of *C. burnetii* antibodies was calculated according to the formula (see below) given by Thrusfield (1995) considering (1) an expected prevalence of 50% (because there were no previous studies to guide us to use a particular prevalence rate), (2) 95% confidence level and (3) 10% desired precision:

$$N={\left(1.96\right)}^{2}\times \frac{P(1-P)}{d^{2}}$$ [Eqn 1]

Where *N* is the number of sample size, *P* is the expected prevalence and *d*^2^ is the absolute precision. Although the calculated minimum sample size was 97 animals, we increased the number of samples to improve the degree of accuracy and to account for some potential sample loss.

Blood samples were collected from 184 camels in 31 herds from January to March 2016. Serum was recovered by centrifugation and stored at −20 °C until analysis.

### Serological testing

Detection of *C. burnetii* antibodies was carried out by using the ID Screen^®^ *C. burnetii* Indirect Multi-species Kits (IDvet, France) following the manufacturer’s recommendations and protocols.

Results were expressed as optical density (OD) and absorbance was read at 450 nm (wavelength) with an EL-800 ELISA plate reader (Biotek Instruments Inc., Winooski, VT, USA). Positive and negative controls (provided by the manufacturer) were used to validate each test.

Samples were considered ‘positive’ if they had an OD value ≥ 40%, ‘questionable’ for values between 30% and 40%, and ‘negative’ for OD value < 30%. These percentages were calculated according to the manufacturer’s instructions. Any sample that initially was classified as ‘questionable’ was re-assayed; after the second assay, any sample that still fell in the OD value of 30% – 40% was assigned as either ‘positive’ or ‘negative’ in an alternating fashion.

The sensitivity and specificity of this ELISA test (100% and 97.8%, respectively; information provided by the manufacturer) were used to convert the apparent seroprevalence to the true seroprevalence using the formula developed by Rogan and Gladen (1978). A herd was considered positive when at least one animal in the herd tested positive.

### Collection of risk factor data

Information regarding potential risk factors was collected at the time of blood sample collection. A structured questionnaire containing 13 variables potentially associated with *C. burnetii* seropositivity was developed using both closed and open-ended questions. Questions pertaining to individual camels included age, breed (Sahraoui, Targui), sex, history of abortion and presence of ticks. Additional data were gathered on general herd and management data such as geographical location of herd, herd size, husbandry system, contact with small ruminants (yes or no), contact with others camel herds (yes or no) and herd size were categorised into three groups: small (< 20 head), medium (20–50 head) and large (> 50 head). The questionnaire was completed by face-to-face interviews with the camel farm owner or manager.

### Statistical analysis

Individual- and herd-level seroprevalence of Q fever was estimated based on ELISA results. Pearson’s chi-square test or Fisher’s exact test was applied to check for significant associations between the potential risk factors and the outcome variables (status of Q fever seropositivity in camels) in a univariate analysis. Multivariate logistic regression was conducted using all variables showing moderate statistical significance (*P* £ 0.25) in a univariate analysis. The logistic regression model was developed in a stepwise forward approach using a likelihood ratio test at each step (with *P* < 0.05 to enter and *P* > 0.10 to exit). Model fit was assessed with the Hosmer and Lemeshow goodness-of-fit test. All statistical analyses were performed using the statistical software SPSS version 22.0 (SPSS Inc., Chicago, IL, USA). In all analyses, two-tailed *P* values < 0.05 were considered as statistically significant.

## Results

Antibodies to *C. burnetii* were found in 71.2% (132/184; 95% CI: 65.2–78.3) of all camels investigated and the true prevalence was calculated as 71.1% (95% CI: 65.1–78.3). The herd-level seroprevalence was estimated at 85.3% (95% CI: 72.8–97.8).

Results for the univariate analysis of individual-level risk factors for *C. burnetii* seroprevalence in camels in southeastern Algeria are summarised in Table 1. Five factors were associated with seropositivity against *C. burnetii*: sex (*P* = 0.013), age (*P* = 0.002), herd size (*P* = 0.012), presence of ticks (*P* = 0.019) and contact with other camel herds (*P* = 0.036). Individual seroprevalence was higher (*P* < 0.05) in females (74.1%) than in males (42.9%) and in camels > 11 years old (85.9%) than in camels < 3 years (47.1%). Seropositivity was greater in camels infested with ticks, whereas there was no difference in seroprevalence of Q fever among breeds (*P* > 0.05). Likewise, geographical region was not predictive of seropositivity against *C. burnetii* among camels. No significant differences were seen for other risk factors, including history of abortion, husbandry system, introduction of newly purchased animals and contact with small ruminants.

**TABLE 1:** Factors associated with animal-level prevalence of antibodies to *Coxiella burnetii* for camel populations of southeastern Algeria.

Factor	Category	*N*	*Coxiella burnetii*				*P*
			Positive		Negative		
			*n*	%	*n*	%	
Geographic location (province, locality)	Ouargla	43	28	65.1	15	34.9	0.683
	Biskra	45	32	71.1	13	28.9	
	El-Oued	42	32	76.2	10	23.8	
	Ghardaia	54	40	74.1	14	25.9	
Sex	Male	14	6	42.9	8	57.1	0.013
	Female	170	126	74.1	44	25.9	
Breed	Sahraoui	143	106	74.1	37	25.9	0.179
	Tergui	41	26	63.4	15	36.6	
Age class (years)	< 3	17	8	47.1	9	52.9	0.002
	3–7	57	36	63.2	21	36.8	
	8–11	39	27	69.2	12	30.8	
	> 11	71	61	85.9	10	14.1	
Herd size (head)	Large (> 50)	128	100	78.1	28	21.9	0.012
	Medium (20–50)	46	27	58.7	19	41.3	
	Small (< 20)	10	5	50.0	5	50.0	
Contact with other camel herds	Yes	180	131	73.3	49	27.2	0.036
	No	4	1	25.0	3	75.0	
Husbandry system	Extensive	122	88	72.1	34	27.9	0.211
	Semi-intensive	24	14	58.3	10	41.7	
	Intensive	38	30	78.9	8	21.1	
Divagation	Yes	89	62	69.7	27	30.3	0.376
	No	95	70	73.7	25	26.3	
History of abortion†	Yes	65	46	70.8	19	29.3	0.411
	No	102	78	76.5	24	23.5	
Introduction of purchased animals	Yes	97	68	70.1	29	29.9	0.603
	No	87	64	73.6	23	26.4	
Presence of ticks	Yes	103	81	78.6	22	21.4	0.019
	No	81	51	63.0	30	37.0	
Source of water	Well	126	91	72.2	35	27.8	0.830
	Lakes/streams	58	41	70.7	17	29.3	
Contact with sheep and goats	Yes	91	66	72.5	25	27.5	0.814
	No	93	66	71.0	27	29.0	

Univariate analyses (*χ*^2^ test for significance).

† , Excludes she-camels < 3 years of age who typically do not reproduce.

Results of the multivariate logistic regression analyses are shown in Table 2. Three variables remained in the final model: (1) age category, (2) herd size and (3) presence of ticks. Seroprevalence increased (*P* = 0.001) progressively with age (OR = 8.81, 95% CI: 2.55–30.41) and seropositivity of camels reared in large herds (74.7%) was higher (*P* = 0.048, OR = 4.46, 95% CI: 1.01–19.59) than that in camels from small herds (42.7%). The odds ratio for the presence of ticks showed that infection was nearly 2.2 times higher in animals with ticks present on their bodies at the time of sampling.

**TABLE 2:** Factors influencing the risk of *Coxiella burnetii* seropositivity among camel populations in southeastern Algeria.

Independent variable	*B*†	Standard error	Odds ratio	95% confidence interval (OR)	*P*
Constant	−1.654	0.899	-	-	0.066
Presence of ticks	0.790	0.363	2.203	1.081–4.490	0.030
Herd size	-	-	8.242	-	0.006
Small (< 20 head)	Reference	-	-	-	-
Medium (20–50 head)	0.318	0.782	1.374	0.297–6.368	0.685
Large (> 50 head)	1.495	0.755	4.459	1.015–19.591	0.048
Age class (years)	-	-	15.362	-	0.002
< 3 (*n* = 17)	Reference	-	-	-	-
3–7 (*n* = 57)	0.672	0.590	1.959	0.617–6.224	0.254
8–11 (*n* = 39)	0.811	0.624	2.250	0.662–7.641	0.194
> 11 (*n* = 71)	2.176	0.632	8.810	2.552–30.413	0.001

Model *χ*^2^ 31.169 with 6 *df*.

Model-2 log likelihood 187.938.

*χ*^2^ goodness of fit = 102.117; *P* value = 0.154.

† , Logistic regression coefficient.

## Discussion

To our knowledge, this is the first investigation on seroprevalence of *C. burnetii* infection in camels in Algeria. This study may therefore represent an important contribution to our scientific knowledge because there is a paucity of published information regarding the status of Q fever in humans and animals in Algeria. The few published epidemiological studies conducted in ruminants were performed in sheep and goats (Khaled et al. 2016; Rahal et al. 2011; Yahiaoui et al. 2013). Clearly, Q fever has been understudied in Algeria. As a consequence of the dearth of published results, the importance of ruminants as *C. burnetii* reservoirs and their role in dissemination of this pathogen are currently unknown.

In this cross-sectional study, the observed individual seropositivity for *C. burnetii* (71.2%) falls within the limits previously reported for camels, which ranged in Ethiopia from a low of 18.6% (Browne et al. 2017) to a high of 90% (Gumi et al. 2013).

Additional published data on Q fever revealed that seroprevalence varied between countries. Prevalence of Q fever antibodies was 28.7% in Iran (Pirouz et al. 2015), 62% and 51.5% in Saudi Arabia (Hussein et al. 2008 and 2015, respectively), 66% in Egypt (Soliman et al. 1992) and 80% in Chad (Schelling et al. 2003). These results clearly established that there is a wide disparity of seroprevalence of *C. burnetii* between camels and other species; the camel seroprevalence is the highest among all ruminant species (Browne et al. 2017; DePuy et al. 2014). Genetic susceptibility of camels to *C. burnetti* infection may explain the high prevalence of coxiellosis in camels (Gumi et al. 2013), but future studies will be necessary to delineate the role of genetic susceptibility as a contributing factor.

In the current study, the high prevalence of *C. burnetii* in camels in southeastern Algeria could be attributed to a number of factors. Ticks are widespread in the Saharian zones of Algeria and they parasitise mainly camels, with a rate of infestation reported at 99.4% (Bouhous, Aissi & Harhoura 2008). The role of ticks as a reservoir of *C. burnetii* has been reported and ticks have been implicated as a major factor in the spread of the infection within humans as well as wild and domestic animals (Asadi et al. 2014; Cantas et al. 2011; Mediannikov et al. 2010; Psaroulaki et al. 2006). In contrast, in other studies performed in ticks collected from animals in some endemic areas in Europe, a very low infection rate of *C. burnetii* was observed which therefore suggests that ticks represent a lower risk of contamination (Astobiza et al. 2011; Sprong et al. 2012). With the identification of new phylotypes of *Coxiella*-like symbionts in a wide variety of ticks (Klyachko et al. 2007; Machado-Ferreira et al. 2016), the differentiation of *C. burnetii* from Coxiella-like organisms remains complex; the relationship between this bacterium and ticks remains a matter of debate and more studies are needed to explore this potential association and the precise mechanism of transmission.

Another potential contributing factor could be that many camels in southeastern Algeria are raised under poor hygienic and sanitary conditions, kept by nomadic herdsmen who follow traditional animal husbandry practices. Moreover, the potential exposure of camels to widespread sandstorm dust contaminated with *C. burnetii* may also contribute to the high-rate infection (Hussein et al. 2015). It is therefore reasonable to expect a higher rate of seroprevalence in camels than in sheep and goats. However, no data on the national (Algerian) disease incidence or on disease surveillance and control efforts for camels presently exist.

The sex of the animals was one of the most important risk factors for seroprevalence of *C. burnetii*. Female animals had higher (*P* < 0.05) seroprevalence than male. Our results are in agreement with previous studies in camels (Abakar et al. 2014; Gumi et al. 2013; Hussein et al. 2015) and in cattle (Carbonero et al. 2015; McCaughey et al. 2010). The high rate of female seroprevalence in our work may be because of the fact that the majority of the old camels are females (92.4% of animals sampled were female) and older females have a greater opportunity to be exposed to *C. burnetii* than younger camels. However, this effect of sex was not true for sheep and goats, because no difference in the seroprevalence of *Coxiella* antibodies was observed between males and females (Asadi et al. 2014). Nevertheless, for humans, the seroprevalence in men was slightly higher than that in women (Anderson et al. 2009; Schelling et al. 2003; Tozer et al. 2011). This effect in humans may be explained by the fact that more men are performing obstetrical work on livestock than women.

No difference in the prevalence of *C. burnetii* among camels in different provinces was noted in our study. This is in contrast to some reports in the literature where Q fever seroprevalence varied widely by geographical locations within the same country and between different countries (Asadi et al. 2014; Hussein et al. 2015; Njeru et al. 2016; Pirouz et al. 2015; Vanderburg et al. 2014). No difference in seropositivity was detected for camels managed intensively versus those managed semi-intensively or extensively.

We did not find any association between history of abortion and seropositivity of camels – a finding also reported by other investigators (Hussein et al. 2008; Wernery & Kaaden 2002). However, this association has been reported frequently in the literature for other species (Asadi et al. 2014). One very recent study indicated that *C. burnetii* was the most prevalent pathogen isolated from uterine swabs collected from camels with a history of reproduction failure (Khalafalla et al. 2017), confirming the implication of this zoonotic organism in uterine infections of dromedary camels. Further studies are necessary to elucidate the role of *C. burnetii* as an abortion-causing agent in camels.

In our study, the risk factors for camel seroprevalence included, in addition to age, large herd size and presence of ticks. These main risk factors associated with Q fever seropositivity were in agreement with those reported in previous studies (Gumi et al. 2013; Pirouz et al. 2015). This result may be explained by the fact that larger herds provide more chances for contact between animals. Our results are in good agreement with previous reports for other species of animals (Alvarez et al. 2012; McCaughey et al. 2010).

This study documented that the serological prevalence among adults aged 11 years and older was eight times higher than that in young camels 3 years of age. Our results are in good agreement with other studies (Gumi et al. 2013; Hussein et al. 2015; Pirouz et al. 2015) which showed that the seroprevalence of *C. burnetii* increased with age. The high rate of seropositivity in older animals could be explained by longer duration exposure to the organism in the environment. Studies on other domestic animals (such as cattle, sheep and goats) have also found the same pattern (Abakar et al. 2014; Alvarez et al. 2012; Gumi et al. 2013; McCaughey et al. 2010). Our findings are also in agreement with studies on humans where the prevalence of antibodies against Q fever increases with age (Psaroulaki et al. 2006; Tozer et al. 2011).

The above findings suggest the presence of ticks as a potential risk factor in the transmission of *C. burnetii* in camels. In line with our findings, previous reports showed that the risk of Q fever infection is greatest in animals with a high rate of tick infestation (Asadi et al. 2014; Cantas et al. 2011; Psaroulaki et al. 2006). In addition, molecular surveys (based on PCR amplification, reverse line blot hybridisation and deoxyribonucleic acid [DNA] sequencing) reported the presence of *C. burnetii* DNA in ticks collected from different domestic ruminants (Aouadi et al. 2017; Kumsa et al. 2015).

Other risk factors (such as breed, husbandry system and contact with small ruminants) potentially associated with seropositivity were not significant in our study. We failed to observe differences as a result of breed in distribution of *C. burnetii* and this result is in contrast with studies that identified breed as a risk factor for *C. burnetii* exposure in cattle (Cantas et al. 2011; McCaughey et al. 2010; Paul et al. 2012) and in sheep and goats (Asadi et al. 2014). Husbandry system (extensive, semi-intensive and intensive management) likewise was not significant in our study – a result that we did not anticipate. Tick infestation was present in all three husbandry systems and we believe that this may be the main reason why husbandry system did not significantly influence *C. burnetii* seropositivity.

Although our original intent in this study was to investigate the potential risk of infection with *C. burnetii* because of quarantine of newly purchased animals, use of disinfectants, treatment of newly purchased animals, movement of animals, camel parity, level of milk production and lactation stage, we could not do so because of a paucity of data.

The choice of serological test for diagnosis is of great importance and may have a remarkable effect in epidemiological studies (Priest & Austin 1993). Some authors consider ELISA to be more specific and sensitive than IFA (indirect fluorescent antibody) for serological surveys of *C. burnetii* (Kittelberger et al. 2009; Soliman et al. 1992). In the present study, however, one potential limitation of our experimental results that should be taken into account is that we evaluated antibodies against *C. burnetii* and did not directly detect antigens. Unfortunately, the ELISA cannot distinguish between active and old infections in animals. Although active infections can be confirmed by detecting *C. burnetii* DNA by using a conventional PCR assay (Boarbi, Fretin & Mori 2016; Muskens et al. 2011), that technology was not available to us during this study.

## Conclusion

Our findings constitute the first known investigation on seroprevalence of *C. burnetii* in camels in Algeria and our results provide strong evidence that Q fever should be considered as a public health and veterinary concern in this country. The prevalence of *C. burnetii* infection in camels is high and widespread in southeastern provinces and camels likely play an important role in the epidemiology of Q fever in the area. Our study revealed that the major risk factors for Q fever seroprevalence in camels are old age, herd size and exposure to ticks. Appropriate measures should be taken to prevent spread of *C. burnetii* and to reduce the risk of Q fever in farm animals and humans in this ecologically and strategically important region of North Africa. Our findings also have direct and substantial relevance to other major camel producing countries of the African continent (e.g. Mauritania, Somalia, Sudan, Mali and Chad) that share common production systems and where camels may serve as a reservoir of the zoonotic agent causing Q fever. Veterinarians and epidemiologists should work together to develop effective strategies for control of this disease.
